# A Systematic Review of risk factors for major adverse cardiovascular events in patients with coronary heart disease who underwent percutaneous coronary intervention

**DOI:** 10.3389/fphys.2025.1514585

**Published:** 2025-04-09

**Authors:** You Zhai, Hongcai Shang, Yan Li, Nan Zhang, Jisi Zhang, Shangwen Wu

**Affiliations:** ^1^ Henan University of Chinese Medicine, Zhengzhou, Henan, China; ^2^ Key Laboratory of Chinese Internal Medicine of Ministry of Education and Beijing, Dongzhimen Hospital, Beijing University of Chinese Medicine, Beijing, China; ^3^ The First Affiliated Hospital of Henan University of Chinese Medicine, Zhengzhou, Henan, China

**Keywords:** coronary heart disease, percutaneous coronary intervention, risk factors, major cardiovascular adverse events, meta-analysis

## Abstract

**Objective:**

This study aims to systematically review the risk factors for major adverse cardiovascular events (MACE) in patients with coronary heart disease who have undergone percutaneous coronary intervention (PCI).

**Design:**

Systematic review and meta-analysis.

**Data sources:**

The Cochrane Library, PubMed, Web of Science, China National Knowledge Infrastructure (CNKI), Wanfang Database, and VIP Database for Chinese Technical Periodicals (VIP) were screened until December 2024.

**Eligibility criteria for selecting studies:**

Case-control studies or cohort studies on the risk factors for MACE in patients with coronary heart disease who underwent PCI. Data extraction and synthesis: The literature review, data extraction, and quality evaluation were conducted by two independent researchers, and the meta-analysis was performed using RevMan 5.4 software.

**Main outcomes:**

The main outcome was that MACE occurred during the follow-up period.

**Results:**

A total of 40 articles were included. The meta-analysis erevealed that dyslipidemia (OR = 1.50; 95% CI [1.19, 1.89], *p* = 0.0007), diabetes mellitus (OR = 1.70; 95% CI [1.43, 2.02], *p* < 0.00001), hypertension (OR = 1.62; 95% CI [1.35, 1.96], *p* < 0.0001), history of smoking (OR = 2.08; 95% CI [1.51, 2.85], *p* < 0.0001), poorer ventricular function (OR = 2.39; 95% CI [2.17–2.64], *p* < 0.0001), impaired left ventricular ejection fraction (LVEF) (OR = 1.86; 95% CI [1.71–2.03], *p* < 0.0001), door to balloon (D-to-B) time (OR = 0.61; 95% CI [0.42–0.88]; *p* = 0.009), thrombolysis in myocardial infarction (TIMI) (OR = 1.41; 95% CI [1.17, 1.70], *p* = 0.0004), renal dysfunction (OR = 1.82; 95% CI [1.37, 2.43], *p* < 0.0001), and multi-vessel coronary artery disease (OR = 0.41; 95% CI [0.37, 0.46], *p* < 0.0001) were significantly associated with MACE after PCI.

**Conclusion:**

The independent risk factors of MACE after PCI are dyslipidemia, hypertension, diabetes mellitus, smoking history, Killip class > II, LVEF ≤40%, D-to-B time >90 min, TIMI flow grade ≤ II, renal insufficiency, and multivessel disease.

## 1 Introduction

Coronary heart disease (CHD) is one of the leading causes of death in the global population (Ananth et al., 2023). Current therapies for CHD include traditional drug therapy, percutaneous coronary intervention (PCI), and coronary artery bypass graft surgery (Baydoun et al., 2019). PCI is currently recommended as the primary revascularization strategy for CHD patients. After PCI, the incidence of major adverse cardiovascular events (MACE) contributes significantly to morbidity and mortality rates (Holm et al., 2023; Liu et al., 2024). A number of studies have shown that within 30 days after PCI, the rehospitalization rates, and the disability rates were very high, which seriously affected the quality of life of patients (Iqbal et al., 2024; Qanitha et al., 2018).

Clinical studies have shown that multiple factors can affect the occurrence of PCI related MACE. The main factors include the following three aspects: 1) Factors related to patients, such as multi vessel disease, atherosclerosis of whole blood vessels, age over 65, low left ventricular ejection fraction (<40%), previous history of myocardial infarction, previous coronary artery bypass grafting, type 2 diabetes, hyperlipidemia, hypertension, chronic or end-stage renal disease and renal failure, anemia, preoperative troponin increase, unstable angina pectoris, and preoperative low-density lipoprotein cholesterol level increase (Madhavan et al., 2020); 2) Factors related to lesions, such as greater saphenous vein bridge vascular disease, eccentric lesions, large plaques and platelet thrombotic burden, plaque rupture; 3) Risk factors related to surgery, such as number of implanted stents, total length of stents, total time of balloon dilation, and total number of balloon dilations (Lin et al., 2023; Nasir et al., 2020; Wang et al., 2020b; Zhang et al., 2024).

Although PCI can effectively reduce the mortality rate, a variety of adverse cardiac events, such as acute heart failure and malignant arrhythmia, may occur after PCI, leading to a poorer long-term prognosis (Kim et al., 2018). The ultimate goal of percutaneous coronary intervention is not only to prolong the patient’s survival but also to improve the patient’s prognosis and quality of life (Head et al., 2018). Multiple clinical studies have demonstrated that MACE occurring within the first year following PCI significantly impact the long-term prognosis of CHD patients (Farshidi et al., 2018; Lu et al., 2023). Current evidence suggests that MACE incidence is associated not only with modifiable lifestyle factors including dietary habits and daily behaviors, but also with established cardiovascular risk factors (Kolkailah et al., 2018). Furthermore, accumulated clinical data indicate that persistent exposure to CHD risk factors continues to influence patient outcomes during the post-PCI recovery period (Khamis et al., 2012). These findings advocate for a paradigm shift toward personalized, multifactorial risk stratification and management in post-PCI care, particularly targeting modifiable factors such as glycemic control and smoking cessation. However, the evaluation indexes are complex, and the results are different. Their reliability and clinical significance need further study. By searching the published literature, this study aims to analyze and explore the risk factors for MACE in patients with CHD after PCI, so as to provide references for the prevention and treatment of MACE after PCI.

## 2 Materials and methods

### 2.1 Search strategy

The meta-analysis was conducted following the Preferred Reporting Items for Systematic Reviews and Meta-Analysis (PRISMA) guidelines (Page et al., 2021). The Cochrane Library, PubMed, Web of Science, China National Knowledge Infrastructure (CNKI), Wanfang Database, and VIP Database for Chinese Technical Periodicals (VIP) were searched from January 2000 to December 2024. The keywords were combined with free words and retrieved manually from foreign medical information resources such as Key Words Platform, the Chinese Journal of Cardiovascular Disease, and the Chinese Journal of Nursing. The Chinese keywords were “coronary angiography, coronary intervention, percutaneous coronary intervention, coronary intervention, and coronary heart disease intervention”. The English keywords were “acute coronary syndrome, coronary atherosclerotic heart disease, coronary heart disease, cardiovascular disease, percutaneous coronary intervention, percutaneous coronary revascularization, coronary angiography, coronary angiographies, percutaneous coronary intervention, percutaneous coronary revascularization,” “risk factors, factor, risk, population at risk, forecasting factor, relative risk, risks,” and “major adverse cardiovascular events, cardiac death, non-fatal myocardial infarction, target-vessel revascularization, stent thrombosis”. Specific search strategies such as PubMed database are as follows: (“Percutaneous Coronary Intervention” [MeSH] OR PCI OR “coronary angioplasty” OR “coronary angiographies” OR “percutaneous coronary intervention” OR “percutaneous coronary” OR “percutaneous coronary revascularization”) AND (“Major Adverse Cardiovascular Events” [MeSH] OR MACE OR “myocardial infarction” OR “cardiac death” OR “stent thrombosis”) AND (“Risk Factors” [MeSH] OR “predictors” OR “prognostic factors”) AND (“humans”{ [Filter]) AND (“2000/01/01” [Date - Publication]: “2023/6/30” [Date - Publication]) AND (English [Language] OR Chinese [Language]).

### 2.2 Inclusion and exclusion criteria of literature

Inclusion criteria were as follows: 1) Case-control studies (CCS) or cohort studies (CS); 2) Publicly published research in Chinese or English; 3) The research subjects were coronary heart disease patients who underwent PCI treatment; 4) The Newcastle Ottawa scale (NOS) scores were ≥7; and 5) The outcome indicators included MACE.

Exclusion criteria were as follows: 1) Repeated publications of literature; 2) Literature published in languages other than Chinese and English; 3) Research without full text, incomplete basic data, or inability to extract data; 4) Literature reviews; 5) The data of odds ratio (OR), 95% confidence interval (CI), and standard error (SE) were not provided, and the data provided could not be converted into OR value, 95% CI and SE; 6) The definition of risk factors not aligned with the European Society of Cardiology (ESC) or the American College of Cardiology (ACC) guidelines; and 7) Patients who had undergone coronary artery bypass grafting.

### 2.3 Literature screening and data extraction

The quality of the literature was evaluated independently by two researchers. Duplicate articles were eliminated, and the title and abstract were read individually according to the inclusion and exclusion criteria. The full texts of the articles were further read for screening. In case of any disagreement, a decision was made through a discussion or consultation with a third party. The data, including the first author, the year of publication, the place of study, the period of study, the type of study, the source of samples, the number of cases in the case group and the control group, and the related risk factors, were extracted. The main outcome was that MACE occurred during the follow-up period.

### 2.4 Methodological quality assessment

The qualities of the chosen literature were evaluated using the NOS. The total score of NOS for CCS or CS was 9 points, where more than 7 points were classified as high-quality literature.

### 2.5 Statistical methods

Review Manager software (version 5.4; the Cochrane Collaboration, Copenhagen, Denmark) was used for meta-analysis. Firstly, heterogeneity was analyzed. If *p* ≥ 0.1 or *I* ^*2*^ ≤ 50%, indicating homogeneity between studies, a fixed-effects model was used for pooled analysis. If *p <* 0.1 or *I* ^*2*^ ≥ 50%, indicating heterogeneity between studies, a random-effects model was used. Furthermore, sensitivity analysis was used to identify sources of heterogeneity. Subgroup analyses were performed when necessary. If there was homogeneity (*p ≥* 0.1, *I* ^*2*^ ≤ 50%) within and between subgroups, the fixed-effects model was used. The random-effects model was used if there was heterogeneity (*p* ≤ 0.1, *I* ^*2*^ > 50%). The results with a *p*-value lower than 0.05 (*p* < 0.05) were considered statistically significant. Publication bias was assessed by funnel plot visual inspection. Sensitivity analysis was used to evaluate the stability of the results by changing the combined model.

## 3 Results

### 3.1 Results of literature search

**FIGURE 1:**
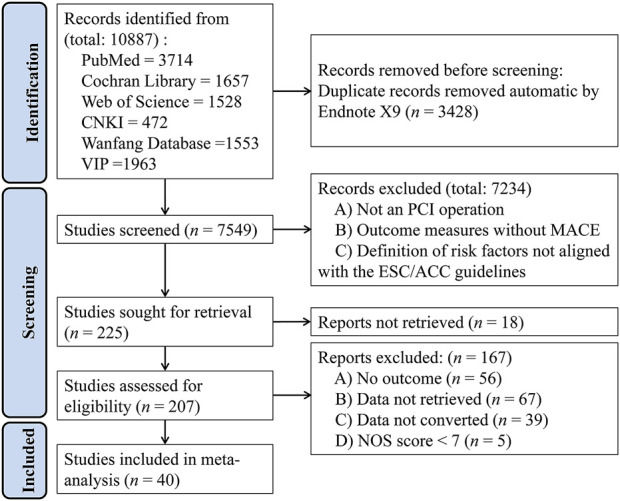
PRISMA (Preferred Reporting Items for Systematic Reviews and Meta-Analysis) flow diagram for studies included in and excluded from the meta-analysis.

A total of 10,887 articles were retrieved, including 1,675 from the Cochrane Library, 3,714 from PubMed, 1,528 from Web of Science, 472 from CNKI, 1,553 from Wanfang, and 1,963 from the VIP database. After removing duplicates and applying the inclusion and exclusion criteria, 40 studies were finally chosen, including 11 in Chinese and 29 in English (Figure 1) (Al-Fiadh et al., 2008; Barthelemy et al., 2015; Batchelor et al., 2020; Birkemeyer et al., 2014; Bufe et al., 2010; Chandrasekhar et al., 2017; Chen, 2016; Elkoustaf et al., 2006; Fan, 2015; Fath-Ordoubadi et al., 2012; Gan, 2017; Gao, 2017; Ghauharali-Imami et al., 2015; Gunnarsson et al., 2024; He, 2016; Hirakawa et al., 2006; Idris et al., 2017; Jackson et al., 2011; Jarrah et al., 2017; Kumbhani et al., 2012; Li et al., 2021; Liao et al., 2015; Lin and Lin, 2024; Ma, 2014; Meller et al., 2013; Motovska et al., 2008; Murphy et al., 2023; Numasawa et al., 2015; Otten et al., 2013; Pendyala et al., 2013; Pu et al., 2011; Toyota et al., 2013; Velders et al., 2013; Wang N. et al., 2020; Wijnbergen et al., 2013; Xu et al., 2022; Yao and Li, 2022; Zanchi et al., 2009; Zhang, 2023; Zimmermann et al., 2009).

The basic characteristics and quality evaluation results of the included literature are shown in Table 1. The total sample size was 117,127. Ten risk factors, each featured in more than three research articles, were included in the final study. All included articles were of high quality with an NOS score of ≥7 (Table 2).

**TABLE 1:** The characteristics of all the included studies.

First author	Year	Type of study	Sample size	Average age	Male (%)	Risk factors
Al-Fiadh,et al. (2008)	2008	CS	2953	62	72.8	①②③
Barthelemy et al. (2015)	2015	CS	1,140	69	76.5	①②③④⑧
Batchelor et al. (2020)	2020	CS	41,137	65	73.2	②④
Birkemeyer et al. (2014)	2014	CCS	1,140	63	74.5	⑤⑦
Bufe et al. (2010)	2010	CS	500	56	75.2	①②③⑦⑨
Chandrasekhar et al. (2017)	2017	CCS	4,851	51	76	④⑧
Chen (2016)	2016	CCS	721	62	66.1	①②③④⑨
Elkoustaf et al. (2006)	2006	CS	1,197	65	68.2	①②③
Fan (2015)	2015	CCS	322	65	71.2	⑤
Fath-Ordoubadi et al. (2012)	2012	CCS	1,641	70	77.3	⑤
Gan (2017)	2017	CS	276	67	-	⑦
Gao (2017)	2017	CCS	152	60	63.8	①②③④
Ghauharali-Imami et al. (2015)	2015	CCS	832	66	73.4	①②③④⑧
Gunnarsson et al. (2024)	2024	CCS	8,364	-	53	③
He (2016)	2016	CS	519	-	53.7	①②⑩
Hirakawa et al. (2006)	2006	CCS	1,036	61	77.3	①②③
Idris et al. (2017)	2017	CCS	3,012	66	72.2	①②③
Jackson et al. (2011)	2011	CS	8,771	63	71	⑥
Jarrah et al. (2017)	2017	CS	2426	63	79.4	⑥
Kumbhani et al. (2012)	2012	CS	1874	66	62.1	⑥
Li et al. (2021)	2021	CCS	778	62	67.8	③
Liao et al. (2015)	2015	CS	701	64	87.3	①②③④
Lin and Lin (2024)	2024	CS	198	72	63	①②③④
Ma (2014)	2014	CS	227	59	79	⑧
Meller et al. (2013)	2013	CS	1,301	54	71.9	④⑤⑦
Motovska et al. (2008)	2008	CS	530	63	70	⑩
Murphy et al. (2023)	2023	CS	789	94	52	②③④
Numasawa et al. (2015)	2015	CCS	10,200	67	79.4	⑥
Otten et al. (2013)	2013	CCS	6,746	61	74	⑤
Pendyala et al. (2013)	2013	CCS	6,929	65	64.3	⑩
Pu et al. (2011)	2011	CS	594	65	75.1	⑨
Toyota et al. (2013)	2013	CCS	4,379	66	72.3	⑦
Velders et al. (2013)	2013	CS	526	72	59	①②③
Wang et al. (2020a)	2020	CCS	3,484	62	75.1	⑧
Wijnbergen et al. 2013)	2013	CS	542	69	74.6	③
Xu et al. (2022)	2022	CS	870	63	76.8	⑨⑩
Yao and Li (2022)	2022	CS	160	64	73.5	②③
Zanchi et al. (2009)	2009	CS	488	63	74	⑦
Zhang (2023)	2023	CCS	94	59	54	①②③④
Zimmermann et al. (2009)	2009	CCS	566	61	71	⑥

Note: ① dyslipidemia; ② diabetes mellitus; ③ hypertension; ④ history of smoking; ⑤ Killip > Grade II; ⑥ left ventricular ejection fraction (LVEF) ≤40%; ⑦ door to balloon time (D-to-B); ⑧ thrombolysis in myocardial infarction (TIMI) blood flow < II; ⑨ renal insufficiency; ⑩ multi-vessel coronary artery disease. CCS: case control study; CS: cohort study; NOS: the Newcastle Ottawa scale.

**TABLE 2:** Quality evaluation results of all the included literature.

First author	S	C	O/E	NOS
Al-Fiadh et al. (2008)	3	2	3	8
Barthelemy et al. (2015)	3	2	2	7
Batchelor et al. (2020)	3	2	3	8
Birkemeyer et al. (2014)	3	2	2	7
Bufe et al. (2010)	3	2	3	8
Chandrasekhar et al. (2017)	3	2	2	7
Chen (2016)	3	2	3	8
Elkoustaf et al. (2006)	3	2	3	8
Fan (2015)	3	2	2	7
Fath-Ordoubadi et al. (2012)	3	2	3	8
Gan (2017)	3	2	2	7
Gao (2017)	3	2	3	8
Ghauharali-Imami et al. (2015)	3	2	3	8
Gunnarsson et al. (2024)	2	2	2	7
He (2016)	3	2	2	7
Hirakawa et al. (2006)	3	2	2	7
Idris et al. (2017)	3	2	3	8
Jackson et al. (2011)	3	2	2	7
Jarrah et al. (2017)	3	2	3	8
Kumbhani et al. (2012)	3	2	3	8
Li et al. (2021)	3	2	2	7
Liao et al. (2015)	3	2	2	7
Lin and Lin (2024)	3	2	3	8
Ma (2014)	3	2	3	8
Meller et al. (2013)	3	2	2	7
Motovska et al. (2008)	3	2	3	8
Murphy et al. (2023)	3	2	3	8
Numasawa et al. (2015)	3	2	2	7
Otten et al. (2013)	3	2	2	7
Pendyala et al. (2013)	3	2	3	8
Pu et al. (2011)	3	2	3	8
Toyota et al. (2013)	3	2	3	8
Velders et al. (2013)	3	2	2	7
Wang et al. (2020a)	3	2	3	8
Wijnbergen et al. (2013)	3	2	3	8
Xu et al. (2022)	3	2	2	7
Yao and Li (2022)	3	2	2	7
Zanchi et al. (2009)	3	2	2	7
Zhang (2023)	3	2	2	7
Zimmermann et al. (2009)	3	2	3	8

Note: NOS: the Newcastle Ottawa Scale; S: selection; C: comparability; O: outcome; E: exposure.

### 3.2 Results of meta-analysis

#### 3.2.1 Dyslipidemia

**FIGURE 2:**
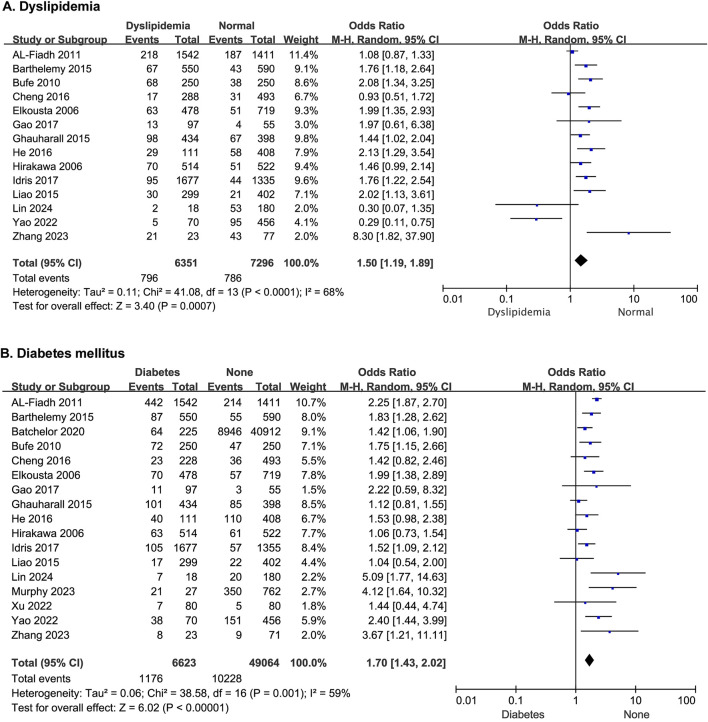
The forest plot depicting the effects of dyslipidemia and diabetes mellitus on MACE after PCI. **(A)** Dyslipidemia **(B)** Diabetes mellitus.

Fourteen studies reported the effect of dyslipidemia on the risk of developing MACE after PCI, and there was no statistical heterogeneity among the studies (*p* < 0.0001, *I* ^*2*^ = 68%). Thus, a random-effects model was used for the meta-analysis. Patients with dyslipidemia exhibited a significantly elevated risk of post-PCI MACE (OR = 1.50; 95% CI [1.19–1.89]; *p* = 0.0007) (Figure 2A).

#### 3.2.2 Diabetes mellitus

Seventeen studies reported the effect of diabetes mellitus on the risk of developing MACE after PCI, with statistical heterogeneity among the studies (*p* = 0.001, *I* ^*2*^ = 59%), but the heterogeneity did not change significantly after a sensitivity analysis. A random-effects model was thus used for the meta-analysis. The results showed that patients with diabetes had a higher risk of MACE after PCI (OR = 1.70; 95% CI [1.43–2.02]; *p* < 0.00001) (Figure 2B).

#### 3.2.3 Hypertension

**FIGURE 3:**
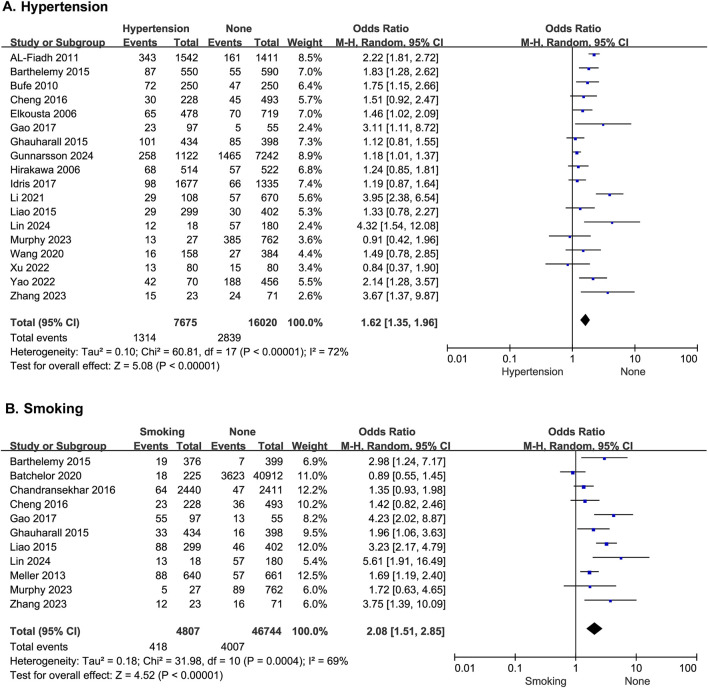
The forest plot depicting the effects of hypertension and history of smoking on MACE after PCI. **(A)** Hypertension **(B)** Smoking.

Eighteen studies reported the effect of hypertension on the risk of developing MACE after PCI, with statistical heterogeneity among the studies (*p* < 0.00001, *I* ^*2*^ = 72%), which did not change significantly after a sensitivity analysis. A random-effects model was therefore used for the meta-analysis. The results showed that patients with hypertension had a higher risk of MACE after PCI (OR = 1.62; 95% CI [1.35–1.96]; *p* < 0.00001) (Figure 3A).

#### 3.2.4 History of smoking

Eleven studies reported the effect of history of smoking on the risk of developing MACE after PCI, with statistical heterogeneity among the studies (*p* = 0.0004, *I* ^*2*^ = 69%), but the heterogeneity did not change significantly after a sensitivity analysis. A random-effects model was therefore used for the meta-analysis. The results showed that patients with a history of smoking had a higher risk of MACE after PCI (OR = 2.08; 95% CI [1.51–2.85]; *p* < 0.0001) (Figure 3B).

#### 3.2.5 Classification of heart function

**FIGURE 4:**
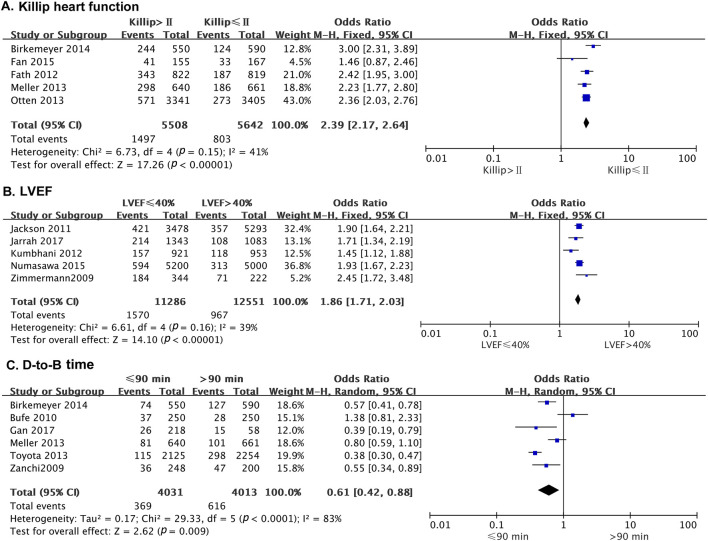
The forest plot depicting the effects of Killip’s cardiac function, LVEF, and D-to-B on MACE after PCI. **(A)** Killip heart function **(B)** LVEF **(C)** D-to-B time.

Five studies reported the effect of heart function on the odds of developing MACE after PCI, with no statistical heterogeneity among the studies (*p* = 0.15, *I* ^*2*^ = 41%). Thus, a fixed-effects model was used for the meta-analysis. The results showed that patients with Killip heart function > II had a higher risk of MACE than those with Killip heart function ≤ II (OR = 2.39; 95% CI [2.17–2.64]; *p* < 0.0001) (Figure 4A).

#### 3.2.6 Left ventricular ejection fraction

Five studies reported the effect of left ventricular ejection fraction (LVEF) on the risk of MACE in patients after PCI. There was no statistical heterogeneity among the studies (*p* = 0.16, *I* ^*2*^ = 39%). A fixed-effects model was thus used for the meta-analysis. The results indicated that the risk of MACE was significantly higher in patients with LVEF ≤40% than in those with LVEF >40% (OR = 1.86; 95% CI [1.71–2.03]; *p* < 0.0001) (Figure 4B).

#### 3.2.7 Door to balloon time

Six studies reported the effect of door to balloon (D-to-B) time on the occurrence of MACE in patients after PCI. There was no statistical heterogeneity among the studies (*p* = 0.37, *I* ^*2*^ = 7%). Therefore, a fixed-effect model was used for the meta-analysis. The results showed that patients with D-to-B time duration >90 min had a higher risk of MACE than those with D-to-B time duration ≤90 min (OR = 0.61; 95% CI [0.42–0.88]; *p* = 0.009) (Figure 4C).

#### 3.2.8 Thrombolysis in myocardial infarction blood flow

**FIGURE 5:**
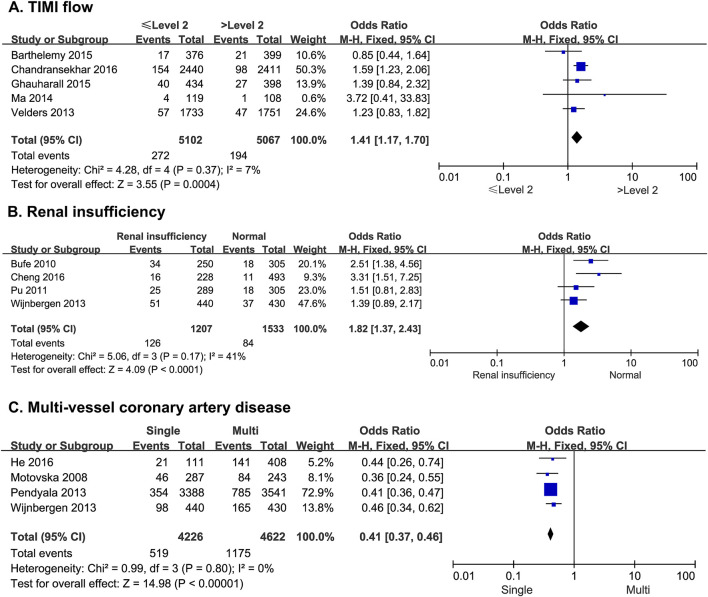
The forest plot depicting the effects of TIMI flow, renal insufficiency, and multi-vessel coronary artery disease on MACE after PCI. **(A)** TIMI flow **(B)** renal insufficiency **(C)** multi-vessel coronary artery disease.

Five studies reported the risk of MACE in patients after PCI with subsequent slow flow and no-reflow. There was no statistical heterogeneity among the studies (*p* = 0.37, *I* ^*2*^ = 7%). A fixed-effect model was therefore used for the meta-analysis. The results showed that thrombolysis in myocardial infarction (TIMI) blood flow ≤ II was significantly higher than TIMI flow > II (OR = 1.41; 95% CI [1.17–1.70]; *p* = 0.0004) (Figure 5A).

#### 3.2.9 Renal insufficiency

Four studies reported the risk of MACE due to renal insufficiency in patients undergoing PCI. There was no statistical heterogeneity among the studies (*p* = 0.17, *I* ^*2*^ = 41%). A fixed-effect model was thus used for the meta-analysis. The results showed that the risk of MACE was significantly higher in patients with renal dysfunction than in patients with normal renal function (OR = 1.82; 95% CI [1.37–2.43]; *p* < 0.0001) (Figure 5B).

#### 3.2.10 Multi-vessel coronary artery disease

Four studies reported the risk of MACE in patients with single-vessel or multi-vessel coronary artery disease after PCI. There was no statistical heterogeneity among the studies (*p* = 0.80, *I* ^*2*^ = 0%). Thus, a fixed-effect model was used for the meta-analysis. The results showed that the risk of MACE was significantly higher in patients with multi-vessel disease than in those with single-vessel disease (OR = 0.41; 95% CI [0.37–0.46]; *p* < 0.0001) (Figure 5C).

### 3.3 Publication bias

**FIGURE 6:**
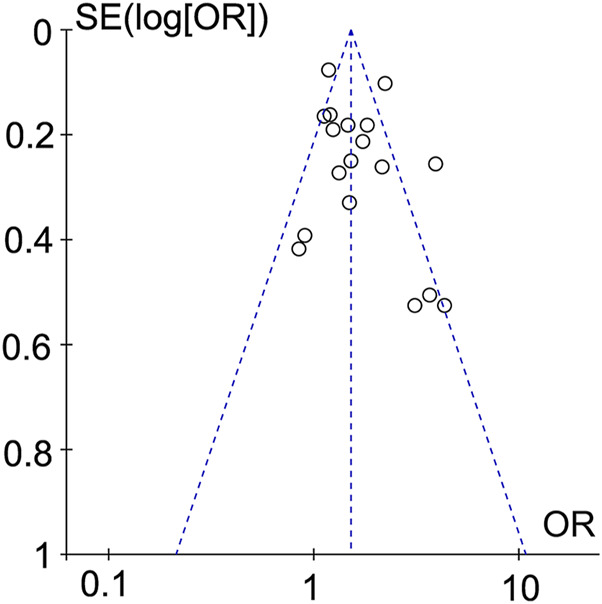
Funnel diagram for dyslipidemia.

Studies that set dyslipidemia as a risk factor were plotted in a funnel plot and subjected to a publication bias test. The results did not show significant asymmetry, suggesting that there was less likelihood of publication bias (Figure 6).

### 3.4 Sensitivity analysis

The results showed that there was no significant difference between the two models, suggesting that the results of this study are stable and reliable (Table 3).

**TABLE 3:** The quantitative combined results of the fixed-effects model and random-effects model.

Risk factors	Fixed effects model		Random effects model	
	OR (95% CI)	*p*	OR (95% CI)	*p*
Dyslipidemia	1.43 [1.27, 1.60]	<0.00001	1.50 [1.19, 1.89]	= 0.0007
Diabetes	1.73 [1.57, 1.90]	<0.00001	1.70 [1.43, 2.02]	<0.00001
High blood pressure	1.51 [1.39, 1.64]	<0.00001	1.62 [1.35, 1.96]	<0.00001
History of smoking	1.88 [1.61, 2.21]	<0.00001	2.08 [1.51, 2.85]	<0.00001
Classification of cardiac function	2.39 [2.17, 2.64]	<0.00001	2.38 [2.07, 2.73]	<0.00001
LVEF	1.86 [1.71, 2.03]	<0.00001	1.85 [1.64, 2.08]	<0.00001
D-to-B time	0.54 [0.47, 0.62]	<0.00001	0.61 [0.42, 0.88]	= 0.009
TIMI blood flow	1.41 [1.17, 1.70]	<0.00001	1.39 [1.13, 1.71]	<0.00001
Renal insufficiency	1.82 [1.37, 2.43]	<0.00001	1.91 [1.30, 2.82]	<0.00001
Multi-vessel disease	0.41 [0.37, 0.46]	<0.00001	0.41 [0.37, 0.46]	<0.00001

Note: LVEF: left ventricular ejection fraction; D-to-B: door to balloon; TIMI: thrombolysis in myocardial infarction.

## 4 Discussion

This meta-analysis of 40 high-quality studies (NOS ≥7) identified dyslipidemia, hypertension, diabetes mellitus, smoking history, Killip class > II, LVEF ≤40%, D-to-B time >90 min, TIMI flow grade ≤ II, renal insufficiency, and multi-vessel disease as independent predictors of post-PCI MACE. The homogeneity of baseline characteristics across studies minimized confounding bias and enhanced the validity of the results.

Metabolic syndromes, characterized by central obesity, hypertension, and dyslipidemia, constitute a cluster of traditional risk factors that synergistically exacerbate coronary artery disease progression. Among them, impaired fasting glucose and hypertension were verified to be associated with a higher risk of MACE after PCI in patients with acute coronary syndrome (Hosseini et al., 2024). Previous studies have demonstrated that the high triglyceride-glucose (TyG) index was associated with an elevated risk of MACE in patients with acute or ST-elevation myocardial infarction undergoing PCI (Köktürk et al., 2024; Luo et al., 2019).

In this study, we also found that dyslipidemia is an independent risk factor for PCI prognosis. Dyslipidemia is characterized by increased levels of triglycerides or low-density lipoprotein cholesterol (LDL-C) and decreased levels of high-density lipoprotein cholesterol (HDL-C), is closely linked to atherogenesis. The LDL-C/HDL-C ratio serves as a reliable marker for assessing coronary artery disease severity in acute coronary syndrome (ACS) patients (Yuan et al., 2024). Elevated LDL-C/HDL-C ratios correlate with higher MACE risk in CHD patients after PCI (Ren and Wang, 2023). The atherogenic index of plasma (AIP), calculated as log (TG/HDL-C), reflects the atherogenic potential of lipoprotein profiles and has been independently associated with MACE (Frohlich and Dobiásová, 2003; Özen et al., 2023). A meta-analysis of 1,055,309 patients confirmed that elevated serum total cholesterol and LDL-C levels increase cardiovascular mortality, whereas higher HDL-C levels exert a protective effect (Jung et al., 2022). Emerging evidence underscores the prognostic value of the ApoB/ApoA-I ratio, which independently predicts 1-year MACE in post-PCI cohorts, potentially reflecting atherogenic lipoprotein imbalances (Zhang et al., 2025). Mechanistically, dyslipidemia drives plaque instability via oxidative modification of small dense LDL particles, which activate NF-κB-mediated endothelial inflammation and foam cell formation (Hasheminasabgorji and Jha, 2021; Higashi, 2023).

Diabetes mellitus is a well-established independent risk factor for MACE following PCI (Latif et al., 2022). Admission glycosylated hemoglobin (HbA1c) levels significantly predict MACE occurrence in diabetic patients undergoing PCI (Bagheri et al., 2024). A large single-center study of 10,724 PCI patients reported significantly higher MACE rates in diabetic compared to non-diabetic individuals (Wang et al., 2018). After PCI of chronic total occlusions of coronary arteries, significant endothelial and smooth muscle dysfunction were present in the distal segments of the successfully recanalized chronic total coronary occlusions (Brugaletta et al., 2012). Higher stress hyperglycemia, a transient elevation of blood glucose, was also reported as a risk factor of MACE (Huang et al., 2022). Diabetes mellitus conferred a 1.7-fold MACE risk (OR = 1.70), primarily attributable to endothelial injury from sustained hyperglycemia (Kaur et al., 2018; Li et al., 2024). Mechanistically, mitochondrial ROS overproduction via NOX4 activation and advanced glycation end product (AGE) accumulation impair nitric oxide bioavailability, while elevated IL-6/TNF-α levels exacerbate vascular inflammation (Knapp et al., 2019; Mordi et al., 2022). These pathways are corroborated by HbA1c’s prognostic value in predicting post-PCI outcomes (Bagheri et al., 2024).

Hypertension significantly contributes to long-term MACE risk post-PCI (OR = 1.62), though its association with in-hospital mortality remains debated (Qi et al., 2023; Saluveer et al., 2017). Prolonged hypertension may promote vascular fibrosis via angiotensin II/angiotensin II type I receptor (Ang II/AT1R) pathway, leading to vascular fibrosis, luminal stenosis, and ventricular remodeling. Long-term elevated blood pressure leads to abnormal vascular wall shear stress, activates NF-κ B pathway, and promotes the release of inflammatory factors (such as IL-6 and TNF- α) (Cao et al., 2022; Elmarasi et al., 2024). The acute blood pressure control during PCI could mitigate immediate risks, explaining the null in-hospital mortality difference observed in some cohorts (Qi et al., 2023).

Smoking history doubled MACE risk (OR = 2.08) in this analysis, consistent with its well-documented cardiovascular toxicity. However, a study revealed that the risk of heart disease is essentially the same as that of non-smokers, after 15 years of smoking cessation (Ahmed et al., 2015). Other paradoxical studies report lower mortality in active smokers post-PCI—a phenomenon attributed to younger smoker demographics or attenuated inflammatory responses (Kumar et al., 2023). Nevertheless, smoking cessation remains critical, as its net cardiovascular harm outweighs transient protective effects.

The Killip classification of cardiac function in patients with acute myocardial infarction (AMI) is considered to be an important index of risk stratification in patients with AMI. Those whose cardiac function is above Killip II grade are considered high-risk. Killip classification ≥ III was an independent predictor of new-onset atrial fibrillation (Shiba et al., 2019). Killip class > I was an independent predictor of MACE in STEMI patients after PCI (Luo et al., 2019). In the present study, we showed that Killip class > II was also an independent risk factor of MACE after PCI. Studies have found that the increased BNP level in Killip II patients reflects the increased ventricular wall tension, activates the cGMP PKG pathway, leads to Ca^2^+ overload of cardiomyocytes and the conversion of energy metabolism from fatty acid oxidation to glycolysis, and exacerbates myocardial stunning (Teragawa et al., 2024).

Consistent with previous reports (Huang et al., 2023b), LVEF was established as an independent risk factor of MACE after PCI in this study. LVEF is an important index for evaluating cardiac ejection function, influencing prognosis and cardiac function. Studies have shown that patients with LVEF <50% have an increased risk of ST (Hu, 2019). Patients with LVEF <50% were independent predictors of 30-day and longer-term mortality for PCI (Mamas et al., 2014). A lower LVEF indicates decreased cardiac output and coronary flow velocity and increased platelet-to-collagen contact, thus increasing the rate of thrombosis.

Prolonged D-to-B time (>90 min) exacerbates ischemic burden by promoting thrombus propagation and microvascular obstruction, thereby attenuating the benefits of timely reperfusion. Thus, the prognosis for patients is often poor (Tran et al., 2017). Shortening the D-to-B time is the key factor in reducing adverse reactions after PCI. After myocardial ischemia for more than 90 min, ATP depletion leads to dysfunction of Na^+^/K^+^ pump, triggering intracellular Na^+^ overload and reverse Ca^2+^ influx, activating mitochondrial permeability transition pore opening, and inducing cardiomyocyte apoptosis (Mastoor et al., 2025).

The TIMI flow grade is the main indicator of myocardial blood perfusion and velocity. A lower TIMI grade is associated with slower blood flow velocity and poorer myocardial perfusion. Tissue microcirculation disturbance is prevalent in patients with low TIMI preoperatively. Even after PCI thrombectomy and vascular dilation, the cardiac blood flow is difficult to recover, which may have adverse effects on the cardiac supply of blood. TIMI ≤ grade 2 indicates coronary microvascular embolism (CME), which is associated with increased platelet neutrophil complex formation and vWF multimer release. The glycocalyx on the surface of endothelial cells in CME area was destroyed, resulting in no reflow phenomenon and expanding the infarct size (Arce et al., 2021). Moreover, elevated levels of hemoglobin and decreased levels of mean platelet volume had a significant association with an advanced grade of TIMI flow in patients who underwent PCI (Parsa et al., 2022). TIMI flow grade I–III is associated with better in-hospital and 1-year outcomes, specifically significantly lower cardiovascular mortality compared to patients who had TIMI flow grade 0 at initial angiography (Shaaban et al., 2022).

Many studies have shown that chronic kidney disease is not only an independent risk factor for cardiovascular morbidity but also significantly influences the prognosis of CHD (Limpijankit et al., 2022; Okina et al., 2021; Polanska-Skrzypczyk et al., 2013). Worsening renal function is an important predictor of mortality in patients with acute myocardial infarction undergoing primary PCI. With the deterioration of the estimated glomerular filtration rate, the short-term and long-term prognosis of patients decreased significantly (Limpijankit et al., 2022; Okina et al., 2021; Polanska-Skrzypczyk et al., 2013). Declining renal function activates NLRP3 inflammasome and promotes IL-18 secretion (Huang et al., 2023a; Thomas et al., 2022). Meanwhile, uremic toxins (such as indole sulfate) induced vascular smooth muscle cell dysfunction and accelerate atherosclerosis (Karbowska et al., 2017; Yu et al., 2022).

Multi-vessel coronary artery disease mainly refers to diffuse lesions involving more than two vessels, which easily cause diffuse myocardial injury, and has become the focus and difficulty in the treatment of CHD. Multi-vessel disease is frequently encountered in primary PCI for myocardial infarction (Akbari and Al-Lamee, 2022; Cho and Nam, 2018). It is still controversial whether complete revascularization is necessary for patients with myocardial infarction and what strategy should be used for the same. Current guidelines for high myocardial infarction recommend that only the infarct-related vessel be addressed during primary PCI unless shock is concurrent (Akbari and Al-Lamee, 2022; Nozoe et al., 2014). Among patients undergoing primary PCI, multi-vessel disease directly indicates a significant increase in postoperative complications, mortality, morbidity, and length of hospitalization (Batra, et al., 2018; Toma et al., 2017). Contrary to expectations, multi-vessel disease was inversely associated with MACE in this study. This paradoxical finding may stem from selection bias, as patients with multi-vessel involvement often receive more intensive surveillance and adjunctive therapies.

This study expands traditional risk stratification models by incorporating dynamic variables such as D-to-B time and TIMI flow, which are rarely included in prior frameworks. Methodological rigor—including dual-blind screening and exclusion of low-quality studies (NOS <6)—enhanced result reliability compared to earlier meta-analyses. The limitations of this study were as follows: 1) The inclusion of only Chinese and English studies may introduce geographic and publication bias, limiting the generalizability of findings to other populations. 2) The number of studies on some risk factors was insufficient to be included, resulting in a lack of data on them in the included studies. 3) The original study included case-control and cohort studies, which lacked high-quality prospective studies and had low demonstration strength.

Based on the above findings, this study proposes the following clinical practice enlightenment to optimize the postoperative management of PCI: firstly, for high-risk patients (such as diabetes mellitus, D-to-B time ≥90 min or multi vessel lesions), it is recommended to dynamically evaluate plaque stability and vascular remodeling through inflammatory markers and coronary imaging 3–6 months after PCI, so as to early identify the risk of restenosis or microcirculation disorders. Secondly, antithrombotic therapy needs to be individualized. Referring to the dual antiplatelet therapy (DAPT) evidence in 2022, the course of treatment for patients with high bleeding risk can be shortened to 6 months, or replaced with ticagrelor monotherapy, so as to balance the risk of ischemia and bleeding. In addition, comprehensive intervention should focus on multiple risk factors: strict control of LDL-C, optimization of blood glucose management, and strengthening smoking cessation support. In the future, a dynamic risk assessment system can be built by combining new markers and artificial intelligence models to promote precise hierarchical management.

## 5 Conclusion

The independent risk factors of MACE after PCI are dyslipidemia, history of hypertension, history of diabetes, history of smoking, Killip class > II, LVEF ≤40%, D-to-B time >90 min, TIMI blood flow ≤ II, renal insufficiency, and multi-vessel disease. During the period of clinical treatment, we should strengthen the control of various risk factors, reduce their influence, and achieve effective control through regular follow-up after discharge. This will significantly improve the prognosis of patients.

## Data Availability

The original contributions presented in the study are included in the article/supplementary material, further inquiries can be directed to the corresponding authors.
